# Embedded, Fully Spray-Coated Pressure Sensor Using a Capacitive Transducing Mechanism

**DOI:** 10.3390/polym10080852

**Published:** 2018-08-01

**Authors:** Christina Offenzeller, Marcel Knoll, Bernhard Jakoby, Wolfgang Hilber

**Affiliations:** Institute for Microelectronics and Microsensors, Johannes Kepler University Linz, 4040 Linz, Austria; marcel.knoll@jku.at (M.K.); bernhard.jakoby@jku.at (B.J.); wolfgang.hilber@jku.at (W.H.)

**Keywords:** pressure sensor, embedded, spray-coated, additive manufacturing, high pressure, high temperature, capacitive, polymer composite, metal/organic

## Abstract

Embedding functional sensor layers directly into mechanical systems in heavy-duty surroundings facilitate the real-time monitoring of the system’s state. This work presents a fully-spray coated pressure sensor that is suitable for applications in the high pressure range. It is embedded into functionalized organic coatings that additionally act as a dielectric for the capacitive sensing mechanism. The sensitivity of the sensor, as well as its long-time stability, has been determined. Additionally, testing has been performed at elevated temperatures to determine the temperature dependent sensitivity that arises from the temperature dependence of the Young’s moduli.

## 1. Introduction

Real-time monitoring of characteristic properties of a mechanical system can be highly advantageous when the wear or failure of a component is investigated. Therefore, including sensors, e.g., for force, temperature or pressure, into mechanical components is a way to prevent the whole system from damage. A very sophisticated way to include such sensors is to embed them directly into the surface of the component, as it is thereby protected against mechanical wear and it moreover measures the quantity exactly at the point of interest.

One of these quantities of interest is the mechanical pressure inside a system. To measure the pressure, there is a multitude of physical effects that can be exploited: Piezoresistive pressure sensors gained importance with the discovery of the piezoresistive effect in silicon [1]. Silicon strain gauges are fabricated directly on silicon diaphragms [2,3] or cantilever beams [4] in order to measure pressure or pressure differences. Piezoelectric materials have been exploited as resonant pressure sensors [5], on cantilevers [6], and even as flexible pressure sensors utilizing piezoelectric polymers [7]. Pressure measurement can also be conducted while using optical methods, by measuring the shift in Bragg wavelength when pressure changes [8] or by combining a diaphragm with a Fabry-Perot setup [9]. It is also possible to measure pressure by measuring the induction current [10] or utilizing magnetostriction [11]. Two major capacitive sensing principles are capacitive diaphragm position sensing [12,13] and the utilization of the elastic properties of dielectric materials in a capacitor [14]. 

This work presents a capacitive pressure sensor that exploits these elastic properties in a plate capacitor. It is less than 15 µm thin and resistant to very high pressures, while consisting of materials that can withstand temperatures of up to 250 °C. Additionally, further processing is enabled as the sensor is designed to be able to remain intact under mechanical wear, i.e., by applying a protective top coat and thereby fully embedding the functional elements. This, in combination with the manufacturing process that consists of spray-coating only, makes the sensor convenient for industrial applications, like condition monitoring inside mechanical systems.

## 2. Sensor Concept and Design

The devised sensor consists of two dielectric layers, a soft coat to increase sensitivity and a rather hard one to make the sensor mechanically stable, on steel substrate and a silver electrode on top, which is again encapsulated in dielectric coating. The mutual capacitance of substrate and electrode is the quantity of measurement as it changes with the pressure that is applied to the electrode. 

**Figure 1:**
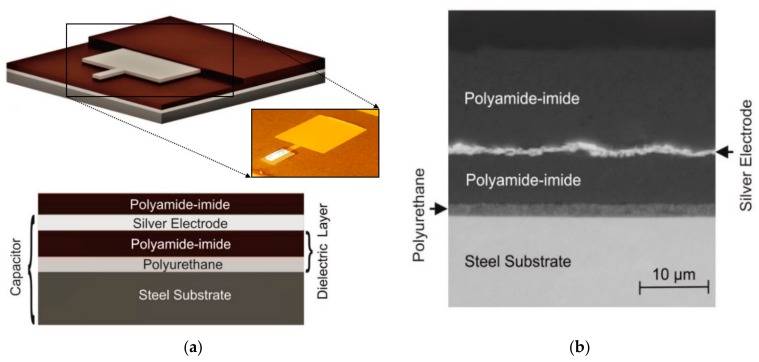
(**a**) The sensor design: A three-dimensional (3D) view (picture and schematic) and a cross section (schematic) which show the layered structure of the sensor, consisting of two conductive layers (steel substrate and silver electrode) and two dielectric layers (polyamide-imide and polyurethane); (**b**) A scanning electron microscopy cross section polish of a fabricated sensor.

In Figure 1a, a schematic of the sensor design is shown: The steel substrate (representing the mechanical component of machine part to be monitored) and the silver electrode form a capacitor with stacked polymer layers in between acting as a dielectric. Figure 1b shows a cross section polish through the sensor layers of a fabricated device taken with a scanning electron microscope. 

It can be seen that the whole setup including the two dielectric layers and the electrode is approximately 10 µm in thickness. The thin layer is the result of spray-coating as fabrication process.

## 3. Materials and Methods

The manufacturing process is composed of several spray-coating steps while using a commercial airbrush (Airbrush Gun AFC-101A by Conrad, Hirschau, Germany). At first, the polyurethane-based coating is thinned with 50 wt % *N*-methyl-2-pyrrolidone and spray-processed onto the steel substrate, which has been pre-heated to 200 °C. After 90 s at 250 °C, the polyurethane-based coating is fully cured and the polyamide-imide-coating can be applied. The polyurethane serves as a softer, more easily deformable, and thereby more pressure sensitive layer, while the polyamide-imide layer has a higher Young’s modulus and it thus prevents the silver electrode from being pushed through the dielectric layer onto the steel substrate at high applied pressures. This polyamide-imide coating consists of 40 wt % *N*-methyl-2-pyrrolidone, 27 wt % p-xylene, and 33 wt % polyamide-imide (Rhodeftal 210 ES by Huntsman Advanced Materials, Salt Lake City, UT, USA). After these components are thoroughly mixed, 0.1 wt % surface additive (BYK 310 by BYK Additives & Instruments, Wesel, Germany) is incorporated to lower the surface tension, which leads to a reduction in pinholes and a lower risk of short circuits. This solution is spray-processed onto the polyurethane-coated steel substrate, which is pre-heated to 170 °C. Subsequently, the polyamide-imide is cured at 250 °C for 30 min.

To pattern the electrode, a high-temperature stable adhesive foil is cut to shape with a cutting plotter and used as a stencil, which is transferred onto the polymer-coated substrate prior to the coating step. To spray-process the electrode material, a silver-nanoparticle paste (KA801 by Dupont, Wilmington, DE, USA) is thinned with 50 wt % ethyl acetate. The polymer-coated substrate is pre-heated to 200 °C for 15 s before the solution is spray-processed onto it. Subsequently, the adhesive stencil is removed and the silver electrode is cured at 200 °C for 20 min. In a final step, the sensor is encapsulated with a polyamide-imide solution in the same way as described before. In order to be able to contact the electrode, a small area of the electrode is covered with the adhesive foil before encapsulating.

To contact the sensor, a copper wire with a diameter of 80 µm was glued to the silver electrode and the steel substrate with a silver-based high-temperature stable epoxy resin (EPO-TEK H20E-8 by Epoxy Technology, Billerica, MA, USA).

## 4. Results

**Figure 2:**
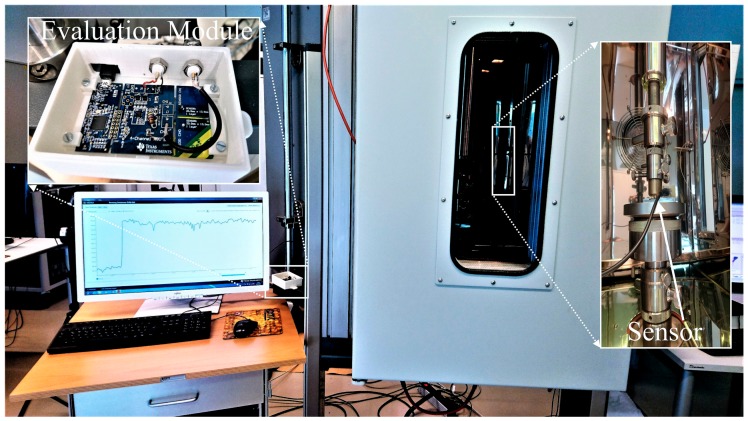
The test setup showing the climate chamber (**Right**) with the pressure sensor clamped in the pressure test bench and the evaluation module (**Left**).

To characterize the manufactured sensor, first the response to changes in pressure at room temperature was investigated. Mechanical pressure was applied using a controlled hydraulic pressure test bench, while the capacitance measurement was conducted with a capacitive sensor evaluation module (FDC2214EVM by TI, Dallas, TX, USA). The test setup is shown in Figure 2.

It consists of a pressure test bench that applies a defined pressure on the sensor. This system is placed in a climate chamber to control the temperature during the measurements. The capacitance evaluation module is placed outside the climate chamber and connected to the PC for recording the measured data.

**Figure 3:**
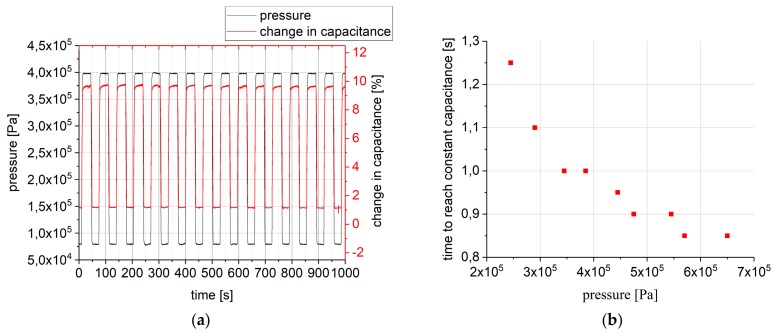
(**a**) The applied pressure (black) and the change in capacitance (red) as a function of time; (**b**) The time the sensor the sensor takes to reach a level of constant capacitance after a pressure has been applied as a function of final pressure level. The initial pressure was 50 kPa for each measurement.

The results of the first measurement presented here are shown in Figure 3a. The change in capacitance is measured as a function of the applied pressure for one hundred cycles at room temperature (25 °C). Each cycle consists of 30 s at 79.5 kPa, followed by 30 s at 397.5 kPa.

On the investigated time scale, the sensor reacts almost immediately to a change in pressure, although it takes some time for the sensor to reach a constant capacitance level, which is due to two phenomena: First, the pressure is not present as a step function, but rather approached by the controller of the pressure test bench. Second, the polymer takes some time to relax into its final state at each pressure level. This time is shown in Figure 3b as a function of final pressure level when starting at 50 kPa. One can see that the time decreases with an increasing final pressure. When the sensor has approached this final state, the capacitance also remains virtually constant when measuring for longer periods.

The change in capacitance is calculated relative to the capacitance at ambient pressure. At the higher pressure levels used, the change in capacitance is in the order of 10%, while at the lower pressure level, it is just slightly over 1%. Assuming a linear dependence between applied pressure and measured capacitance signal, this results in an averaged sensitivity of 0.03% relative capacitance change per kPa.

**Figure 4:**
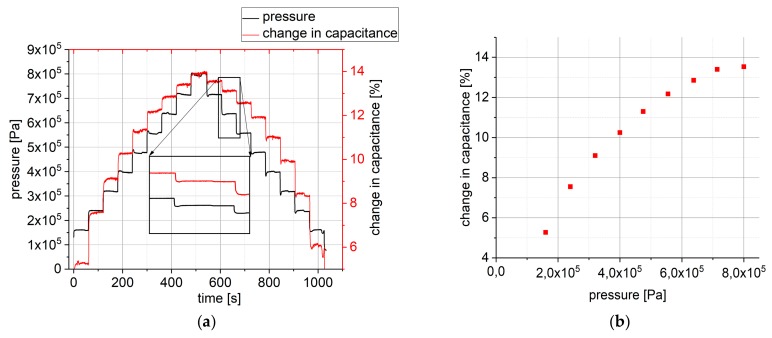
(**a**) The pressure (black) and change in capacitance (red) as a function of time. Each pressure level is held for one minute; and (**b**) Change in capacitance at each pressure level.

However, in reality, this change is not independent from the applied pressure level, as can be seen in Figure 4. In the measurement that is shown in Figure 4a, the pressure (black) is ramped up in steps of 79.5 kPa from 159 to 795 kPa and then ramped down with the same step size, again at ambient conditions (25 °C). Each pressure level is held constant for one minute. It can be seen that, while the pressure step size remains constant, the change in capacitance at each pressure change decreases with increasing pressure levels. In Figure 4b, this is illustrated as the change in capacitance from lower to higher pressure relative to the unloaded sensor as a function of lower pressure level. For pressures that are in the range above 1 MPa, the sensor will detect almost no change in capacitance anymore. 

A second observation which can be made in Figure 4a is that, at the same pressure level, the capacitance levels while ramping down are not the same as while ramping up the pressure. After once loading with a certain maximum pressure, however, the response to a pressure below this maximum pressure is again constant over many cycles (as in Figure 3a). This might be due to the fact that the polymer rearranges under pressure and after this rearrangement process is completed, the sensor output becomes stable up to the maximum pressure that it was loaded with before.

**Figure 5:**
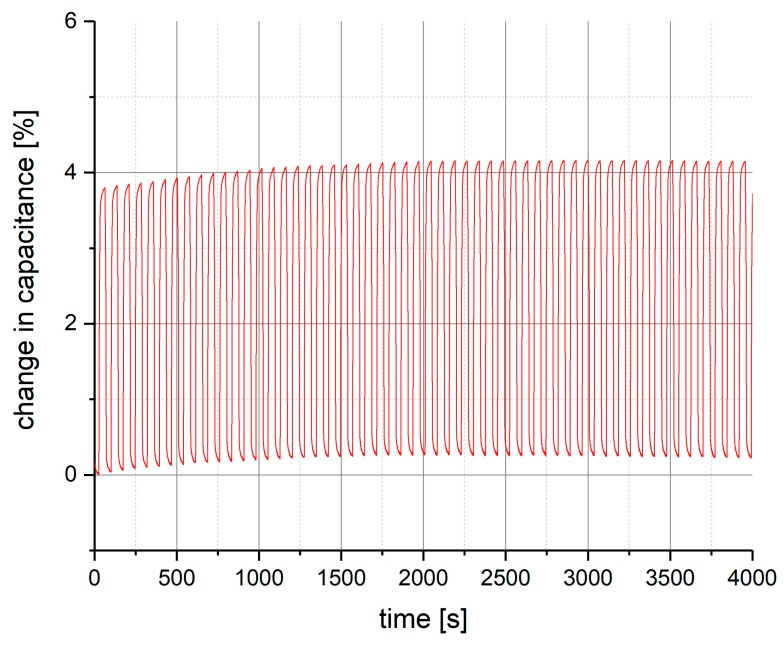
The change in capacitance as a function of time for the first hour of a long-time measurement cycle.

For pressures below 1 MPa however, the manufactured sensor produces a very stable output. This has been verified in a long-time measurement cycle over several hours at ambient conditions (25 °C), in which pressure has been applied to the sensor in periods of high pressure, followed by periods of low pressure. In Figure 5, the results of the first hour of this measurement cycle are shown, after that, no more changes in the measurement signal have been observed. It can be seen that, at the first use of the fabricated sensor, there is a set in phase, in which the change in capacitance increases with each pressure cycle. After the first 30 min, however, the sensor signal remains stable for the rest of the measurement.

**Figure 6:**
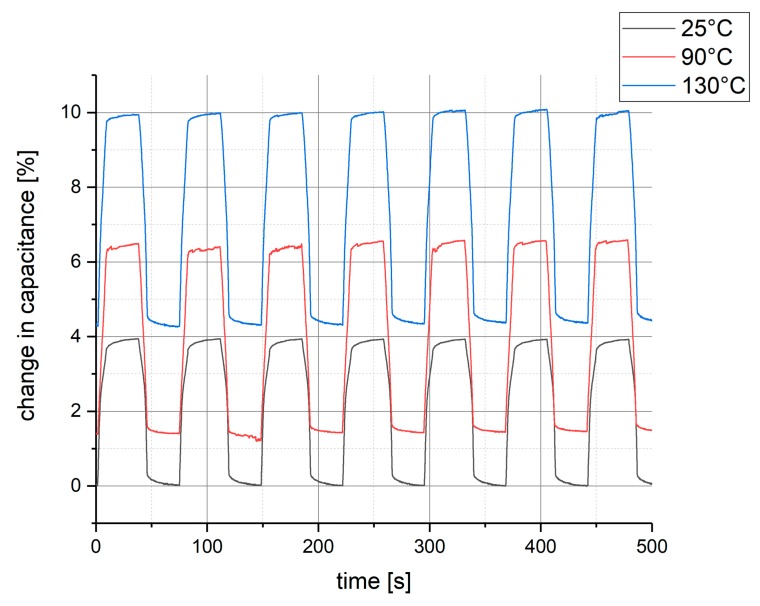
The change in capacitance as a function of time with pressure steps applied at three different temperatures: 25 °C (black), 90 °C (red) and 130 °C (blue).

The same measurement, as shown in Figure 5, has also been conducted under elevated temperatures in a climatic chamber to test for temperature dependence. As can be seen in Figure 6, there is a rather strong influence of the temperature on the sensor signal that needs to be compensated in a measurement application.

The results shown in Figure 6 is the sensor response to pressure steps at three different temperatures: 25 °C (black), 90 °C (red), and 130 °C (blue), which have been held constant during each measurement cycle, respectively. Due to a limitation in the test setup, 130 °C is the highest temperature at which testing was possible, although the materials are stable up to 250 °C. The applied pressure alternates between 10 Pa and 1.3 kPa. As one can see, the temperature affects the sensor in two ways: First, the capacitance at the lower pressure level increases with increasing temperature, as the dielectric coatings soften. Second, the change in capacitance from low to high-pressure level increases with an increasing temperature. At room temperature, this change is approximately 4%, while at 90 °C, it is already 4.7% and at 130 °C 5.8% change. Again, this is due to the fact that at higher temperatures the dielectrics soften and the electrodes distance decreases stronger under pressure.

## 5. Discussion

In this work, a pressure sensor is presented that has been manufactured exclusively by spray-coating onto a metallic substrate (e.g., a machine component to be monitored). This manufacturing process—combined with the mechanically stable design and the high-temperature stable materials—makes it suitable for industrial applications that also involve further component processing. 

The manufactured sensor has been tested regarding its output signal and the corresponding long-time stability. The response of the sensor is fast and stable over long periods of time. However, the sensor output is not independent from the initial pressure level, and is therefore not linear with pressure. A calibration is needed to determine the change in capacitance as a function of pressure. Additionally, the sensor has been characterized regarding the temperature dependence of its output. The results show that the temperature has a strong influence on the sensor response. In comparison to the change in capacitance at room temperature, the response at 90 °C is 17.5% higher, and at 130 °C the increase in response is 45%. This makes the sensor more sensitive at elevated temperatures. If the sensor is to be used in surroundings of varying temperature, the temperature effect needs to be compensated, e.g., by combining it with an embedded temperature sensor [15]. For use at conditions of relatively stable high temperatures and high mechanical pressures, as are present in many mechanical processes, e.g., in heavy industry or automotive production, this sensor enables the stable measurement of pressure inside the mechanical system exactly at the point of interest without the need for expensive manufacturing processes.
